# In artificial roost comparison, bats show preference for rocket box style

**DOI:** 10.1371/journal.pone.0205701

**Published:** 2018-10-31

**Authors:** Julia P. S. Hoeh, George S. Bakken, William A. Mitchell, Joy M. O’Keefe

**Affiliations:** 1 Center for Bat Research, Outreach, and Conservation, Indiana State University, Terre Haute, Indiana, United States of America; 2 Department of Biology, Indiana State University, Terre Haute, Indiana, United States of America; Institute of Zoology, CHINA

## Abstract

Understanding microhabitat preferences of animals is critical for effective conservation, especially for temperate-zone bats, which receive fitness benefits from selecting optimal roost microhabitats. Artificial roost structures are increasingly being used in conservation efforts for at-risk bat species. To evaluate microhabitat differences in common artificial roost structures and determine if roost selection occurs based on structure type, we installed artificial roosts of three different styles (bat box, rocket box, and bark mimic) in six clusters. We compared size and measured temperature parameters (12 points/roost) while bats were excluded from one cluster. We simultaneously conducted census counts during the active season at five more clusters open to bats for 1–2 years. The rocket box style provided larger entrance area, surface area, and volume versus other roost types. Microclimate varied with roost design. More positions inside the bat box and rocket box stayed within critical temperature limits for bats (0–45°C)—i.e., were usable. The bark-mimic provided less usable space than the rocket box and, often, large proportions of the roost were > 45°C_._ The rocket box provided the widest temperature availability in a given hour (max range available 7°C) and was more stable than the bark mimic. A maternity colony of Indiana bats (*Myotis sodalis*) selected the rocket box style; four of five available rocket boxes became primary maternity roosts, with 2–210 bats emerging per night. Future work should aim to manipulate roost size, temperature availability, and temperature stability in isolation to identify which features drive roost microhabitat selection by bats. Comparative studies of artificial roosts account for some inherent irregularity in natural systems, allowing us to study the dynamics of roost microhabitats. We recommend season-long monitoring of microhabitat in novel artificial refuges and comparative studies of artificial and natural roosts, and urge managers to consider potential positive and negative effects when substituting artificial roosts for natural habitat.

## Introduction

Optimal microhabitats provide fitness benefits [1–3] and, thus, understanding microhabitat preferences could be critical to implementing effective conservation and management strategies for animal populations in peril. Availability of optimal roosting habitat may limit bat populations in the temperate zone, where bats select roosts that facilitate pup rearing, energy conservation, social interactions, and predator avoidance [4]. During summer, when female bats gather in colonies to rear pups, energy conservation is an important selective pressure governing roost preferences [2,5]. Temperate-zone bats are small-bodied [6], have energetically costly movement [7], and rely on fluctuating food resources [8], leading to a delicate balance between energy intake and expense. Bats use torpor to facilitate this balance. Entering torpor reduces the energetic costs of maintaining a constant body temperature by reducing energy expenditure, water loss, and other physiological costs [9]. Non-reproductive females or male bats may be more likely to use torpor than pregnant or lactating bats and thus use roosts that facilitate torpor bouts [10,11]. However, cold temperatures can force bats to use torpor even when it is not reproductively advantageous—e.g., longer torpor bouts during pregnancy and lactation delay embryo and neonate development [12,13]. In contrast, excessive heat might force bats to expend energy moving to cooler positions [14] or could be fatal [15]. Use of suboptimal roosting microhabitat or loss of a high-quality roost is linked with lower reproductive success [1] and, thus, female bats should select roosts with favorable microhabitats.

There is substantial evidence that temperate-zone cavity- or crevice-dwelling bats select roosts based on thermal and size characteristics. In building roosts, a maternity colony of soprano pipistrelles (*Pipistrellus pygmaeus*) selected warm positions with a maximum temperature of 40°C, reducing costs of maintaining normothermy or forced torpor [16]. In forests, bats in maternity colonies generally select tree roosts that are large diameter, in early- to mid-decay, often taller than surrounding trees, and with high solar exposure [17,18], all characteristics that frequently result in warmer roosts. Crevice-dwelling species, such as Indiana bats (Vespertilionidae: *Myotis sodalis*; [18]), likely favor warmer maternity roosts that allow for passive rewarming after torpor bouts and roosts with more roosting volume or surface area to accommodate large social groups. Unfortunately, important roost characteristics are often correlated, making it difficult to isolate the critical factors driving roost habitat selection. Further, it is difficult to measure microclimate inside natural roosts because roost tree presence is often highly irregular at multiple spatial scales and microhabitats are typically inaccessible for study (e.g., bark patches or cavities on dead trees).

We can reduce some of the uncertainty regarding roost habitat preference by comparing bat use and microhabitat characteristics of replicated, structurally-specified artificial roosts of different designs. Various temperate-zone bat species use a wide array of artificial roost styles, likely presenting varied microhabitats—e.g., large, freestanding structures that accommodate thousands of bats [19], small boxes designed to mimic cavity roosts [20], and bark- and crevice-roost mimics (e.g., rocket box, [21]; bark-mimics, [22–24]). When assessing microhabitats, it is important to exclude bats for microclimate measurements, unless specifically considering the structure’s metabolic heat retention, as number of bats in a roost affects the internal temperature [25,26]. Although we can control bat presence and other factors in artificial roosts, there are relatively few studies comparing microhabitats in various artificial roost styles (see review, [27]). Despite our limited knowledge, resource managers are deploying a variety of artificial structures to provide bat habitat and evaluating success based solely on use by bats (e.g., [22,28,29]). Artificial roosts are often deployed without comparative evaluations of features that may contribute to selection (but see, [23,30,31]).

We conducted an artificial roost selection experiment at a site occupied by a maternity colony of Indiana bats. Our goal was to determine if microhabitat differences were present in three artificial roost types commonly used in habitat restoration (bat box, rocket box, and bark mimic) and if bats showed preferences by roost type. We installed these roosts in clusters with one of each style, and placed six clusters across the landscape. For the three styles, we described: (1) physical characteristics, including volume, surface area, and entrance area; (2) detailed temperature parameters with bats excluded; and (3) evidence for selection by Indiana bats. We identified physical and thermal characteristics differing among the three styles and delineated key areas for future research.

## Methods

### Study area

We conducted this study in a highly fragmented landscape at an urban-rural interface southwest of Indianapolis, Indiana, U.S.A. (39°39’N, 86°20’W). A colony of Indiana bats showed annual fidelity to the ~1045 ha area [32]. Agriculture, housing, warehouses, and major transportation corridors dominated most of the landscape. Centered along a medium-sized, permanent stream, the East Fork of White Lick Creek, were protected lands, comprised of residual woodlots (most trees < 120 years old, [33]), replanted woodlots, and restored wetlands. In 1992, researchers began monitoring the bat population and installing artificial roosts [34]. Male Indiana bats first used artificial roosts at the site in 1995 [24], but the maternity colony of 100–200 adult females and their pups [35] was not detected using artificial structures until 2003 [28]. Due to the bats’ history of artificial roost use, this was a logical site to assess use of newly installed structures.

We measured roost temperatures from March to September 2016, and roost selection by bats during the active seasons of March to October 2015–2016. We recorded air temperature at 10-minute intervals from 25 May–20 October 2016 with a portable weather station (Onset Computer Corporation Inc., Model H21-002 HOBO Micro Station, Bourne, MA). We obtained daily precipitation and daily mean cloud cover from a weather station 9 km from the field site (KIND station, NOAA National Climatic Data Center). From this station we also obtained air temperature for 21 March–25 May 2016, prior to installation of our portable weather station. In 2015–2016, this area averaged 110 cm of annual precipitation, and active season (March–October) air temperatures ranged from -17–34°C.

### Comparing roosts

#### Description of roost styles and placement

**Fig 1:**
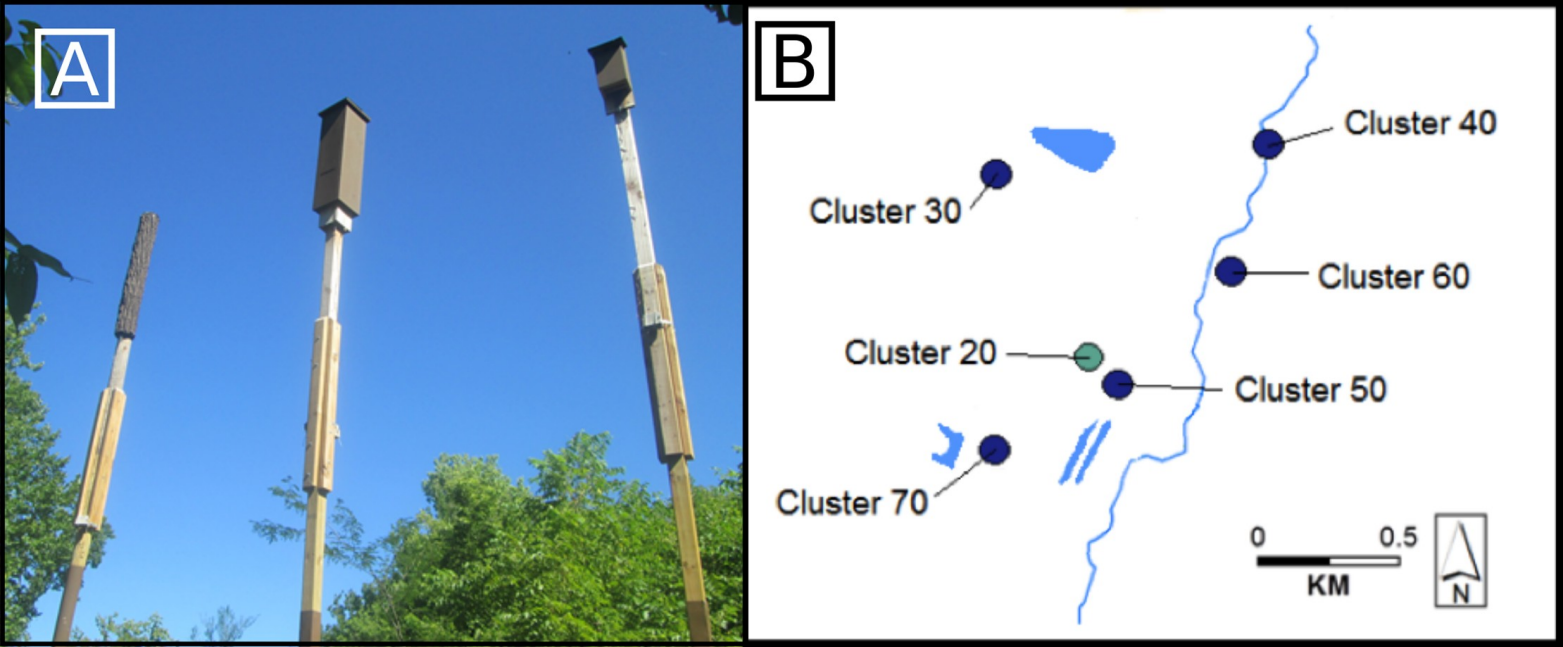
Photo of clustered roosts and map of roost clusters installed at site. (A) One of six artificial roost clusters installed near Plainfield, IN. Each cluster contained one bark mimic (left), one rocket box (center), and one bat box (right) placed 2 m apart and randomly installed in a west to east line. (B) Map of roost clusters; dark gray indicates the roosts in this cluster were open to bat use, light gray indicates bats were excluded from roosts in the cluster for detailed temperature recording. Clusters 30, 40, and 50 were installed in 2015 and clusters 20, 60, and 70 were installed in 2016.

We installed three artificial roost styles: 3-chambered traditional birdhouse style bat boxes (bat box), 2-chambered rocket boxes (rocket box), both made from untreated and planed pinewood, and bark-mimic roosts (bark mimic; Fig 1; Tables 1 and 2) made from polyurethane elastomeric bark material. We mounted all roosts on posts with the roost top at 6 m. Tree roosts for Indiana bats average 8.6 m in height [36], though height relative to neighboring trees may be more important than absolute height [18]. To ensure the three styles were comparable with respect to mounting post dimensions, we cut a bark sheet (130 × 100 cm, full size) in half lengthwise (130 × 50 cm, half size) to fit the circumference of a 12-cm diameter post rather than wrapping a full sheet around a 30-cm pole as specified by the distributor [22].

**Table 1:** Terms, abbreviations, and factors as defined for a study of artificial bat roosts near Plainfield, IN.

Term	Definition
Bat box	3-chambered traditional birdhouse style bat box
Rocket box	2-chambered rocket box
Bark mimic	Modified BrandenBark bark-mimic roost
Cluster	Group of three artificial roosts, one each of bat box, rocket box, and bark mimic
T_CR_	Critical temperature thresholds for temperate-zone bats, defined as 0°C and 45°C
T_mean_	Daily (00:00–23:59) air temperature mean (°C)
T_range_	Daily air temperature range (°C)
Cloud cover	Daily cloud cover mean (%)
Precipitation	Daily precipitation (cm)
Proportion usable	Proportion of temperature data loggers (11–12/roost) that stayed within TCR in a day
Availability	Average hourly range (max−min) of temperatures available within each roost as recorded by temperature data loggers (11–12/roost)
Variability	Difference between the single daily maximum and single daily minimum temperature recorded by any temperature data loggers within each roost (11–12/roost)

**Table 2:** Characteristics of three adjacent artificial bat roosts near Plainfield, IN from which bats were excluded March–September 2016.

Roost characteristics	Bat box	Rocket box	Bark mimic
Material	Wood	Wood	Polyurethane
Air vents	No	Yes	No
Previously used by Indiana bats	Yes^a^	Yes^b^	Yes^c^
Num. of chambers	3	2	1
Height (cm)	40	107	130
Width/diameter (cm)	18	26	16
Roosting surface area (cm2)	3,957	23,217	10,504
Entrance area (cm2)	100	223	72
Volume (cm3)	3,100	17,700	3,200

Rocket box provided the greatest surface area, entrance area, and volume.

^a^[28]

^b^[29]

^c^[22]

Before installation, we measured internal volume (cm^3^), roosting surface area (cm^2^), and entrance area (cm^2^) in each roost type. We filled one of each style with dry corn kernels to measure approximate volume (we chose corn because it was unlikely to spill through gaps and easy to remove prior to installation). We calculated roosting surface area as dimensions of the inside of the wood or bark-mimic sheet minus areas inaccessible due to roost design, and entrance area as the two-dimensional space at the base of the roost through which bats could enter (nearest cm). The rocket box had > 2 times the entrance and roosting surface area, and > 5 times the volume of the next closest roost type (Table 2). The roosting surface area of the bark mimic was > 2.5 times that of the bat box, but the bat box and bark-mimic roost were similar in volume (Table 2).

We installed roosts in six clusters (with one of each roost type, Fig 1) on the southern edge of wooded areas, with no canopy cover above. The bat box and primary entrance of the bark-mimic roost faced south. We installed rocket boxes with the two exterior vents (each 15 × 1 cm and 30 cm from the bottom) facing north and south. We took detailed temperature measurements in one bat-exclusion cluster (openings were covered with hardware cloth, allowing airflow).

#### Temperature in bat-exclusion roosts

In the bat-exclusion cluster, we measured temperature (21 March–7 September 2016; 170 days; n = 71,680 temperature data points, in total) of each artificial roost style. We installed 12 temperature data loggers inside each roost (Thermochron iButton, Maxim Integrated, San Jose, CA; 0.5°C increments with accuracy ± 1°C). At the top, middle, and bottom, four thermochron data loggers were enclosed in mesh bags and attached to the interior roost wall at four intercardinal directions (southeast, southwest, northwest, and northeast). Because our primary goal was to determine the maximum temperature ranges and we had only a limited number of thermochrons, we did not measure temperatures in the rocket box’s inner chamber, which may be more stable than the outer chamber. Thermochrons recorded every 2 hr, with half recording on even and half on odd hours such that roost temperature was recorded every hour across the survey period (4,072 hours). The thermochron in the middle northeast of the bark-mimic roost failed to record; we could not correct this error and, thus, had only 11 thermochron points for this roost. We also measured humidity at three points in each roost using an iButton Hygrochron (Maxim Integrated), which uses a capacitive polymer sensor (typical relative humidity accuracy is ± 3.5%); there was little to no variation among roosts [37].

With respect to temperature, we evaluated proportion usable, availability, and variability for each roost in the exclusion cluster (Table 1). Proportion usable was the proportion of all thermochrons in each roost that stayed within T_CR_ in a 24-hr day (Table 1). We assumed Indiana bats could use portions of the roost that remained within the limits of upper and lower critical temperatures (T_CR_) for temperate-zone bats, defined here as 0 and 45°C. At < 0°C, a bat must expend energy to maintain a torpor threshold and eventually arouse to rewarm [38–40], whereas temperatures > 45°C are fatal after 1 hour of exposure in laboratory settings [41,42]. We assumed bats were unlikely to change roosts during the day [43] and that space limitations might hinder bats from repositioning within a full roost. Thus, if a thermochron registered outside T_CR_, that portion of the roost was considered unusable for the entire day. Availability was the mean range of temperature available each hour of the day (Table 1). To determine this value, we first calculated the roost temperature range at each hour of the day using data from all active thermochrons (e.g., if temperature ranged from 15–17°C at 9:00 am, the instantaneous range was 2°C). We then calculated mean instantaneous range for each 24-hr day. This represented the daily temperature availability, or how many degrees of temperature a bat could select from during the day. Variability was the daily (24 hr) roost temperature range, or difference between the single daily maximum and minimum temperatures recorded by any thermochron (Table 1). A wider daily range indicated the roost was more variable and a narrower range indicated less variation (i.e., daily temperatures were more stable).

### Assessing bat preference

Five of the six clusters referenced above were open to bat use, three installed in 2015 and two more added in 2016 (Fig 1). These five bat-selection clusters were installed within 1.0 km of the exclusion cluster and 0.5–1.5 km of each other. At the bat-selection clusters, we conducted emergence counts and spotlight checks at least two times per week from mid-March to mid-October, 2015 and 2016. Daytime spotlight checks were conducted with binoculars and lights (≥ 1,000 lumens) directed into the roost. If number of roosting bats could not be determined via spotlight, we returned to conduct an evening emergence count (30 min before sunset until 30 min after sunset or 10 min after the last bat emerged), when feasible. We conducted a combined 749 emergence and spotlight counts at three bat-selection clusters in 2015 (9 roosts; mean of 83 counted days/roost) and 1,465 counts at five bat-selection clusters in 2016 (15 roosts; mean of 98 counted days/roost). We counted 0–210 bats emerging from clustered roosts. Research protocols were approved by Indiana State University's Institutional Animal Care and Use Committee (IACUC 559972–1), and followed guidelines from the American Society of Mammalogists [44] and a federal recovery permit held by J.M. O'Keefe (TE206872).

While we have observed northern long-eared (*M*. *septentrionalis*) and big brown bats (*Eptesicus fuscus*) using artificial roosts at our site, we are confident the majority of bats in bat-selection clusters were Indiana bats. We tracked 17 Indiana bats to these roosts in 2015 and 2016 [45,46]. Other evidence includes year-to-year roost fidelity by Indiana bats (i.e., use of two clusters in both 2015 and 2016), timing of colony shifts across the landscape [45,46], and DNA confirmation of guano pellets collected from guano traps (1 m^2^ portion of mesh screening) suspended at the base of all 15 bat-selection roosts [37]. During spotlight checks, we detected a maximum of 1–2 big brown bats per roost (0.2% of total observed bats) and no northern long-eared bats.

### Data analysis

We conducted all statistical analyses using R version 3.1.2 [47]. We examined the normality and homogeneity of variances before applying parametric statistics. We assessed significance at α = 0.05 and present means ± SE unless otherwise noted. We did not test for differences among mean temperatures by position or roost type, as positions are not directly comparable due to differing roost characteristics and mean values obscure subtle differences important for energy balance. However, we note that means may be important for developing cost functions to assess roost optimality [48].

We conducted a beta regression (package betareg, [49]) to assess proportion of exclusion roosts that stayed within the TCR. Proportion usable was first transformed to values between 0 and 1 [50], then regressed with these main effects: roost type, day length (in minutes), and four daily weather variables defined in Table 1 [air temperature mean (T_mean_, °C), air temperature range (Trange, °C), cloud cover mean (cloud cover, %), and precipitation (cm)]. We discarded non-significant main effects (based upon *Z* values) and evaluated interaction terms only for significant main effects.

We used analysis of covariance (ANCOVA) to assess the effects of box type and daily weather parameters (T_mean_, Trange, precipitation, and cloud cover) on roost temperature variability and availability. We initially included all main and interaction effects, but during model simplification we removed all non-significant parameters (based on F values) and any interactions not involving box type, as we aimed to describe difference among roost types. For predictor variables that significantly interacted with box type, we present interaction plots of the response and predictor variable by box type, with regression lines and confidence intervals based on those interactions, rather than the full model.

To determine bat preference by roost type, we calculated the total bat days, defined as one bat using the roost on one day, summed across the season. We also determined the maximum number of bats observed in one night at a roost and designated roosts as primary (≥ 30 bats emerging in one night, [51]) or not. Additionally, to determine whether box type affected maximum weekly counts, we fit a generalized linear mixed effects model (GLMM) by maximum likelihood with Laplace Approximation (lme4 package, [52]). From residual versus fitted plots, we selected a negative binomial distribution for the GLMM, specifying cluster as a random effect and box type as a fixed effect.

## Results

### Temperature

**Fig 2:**
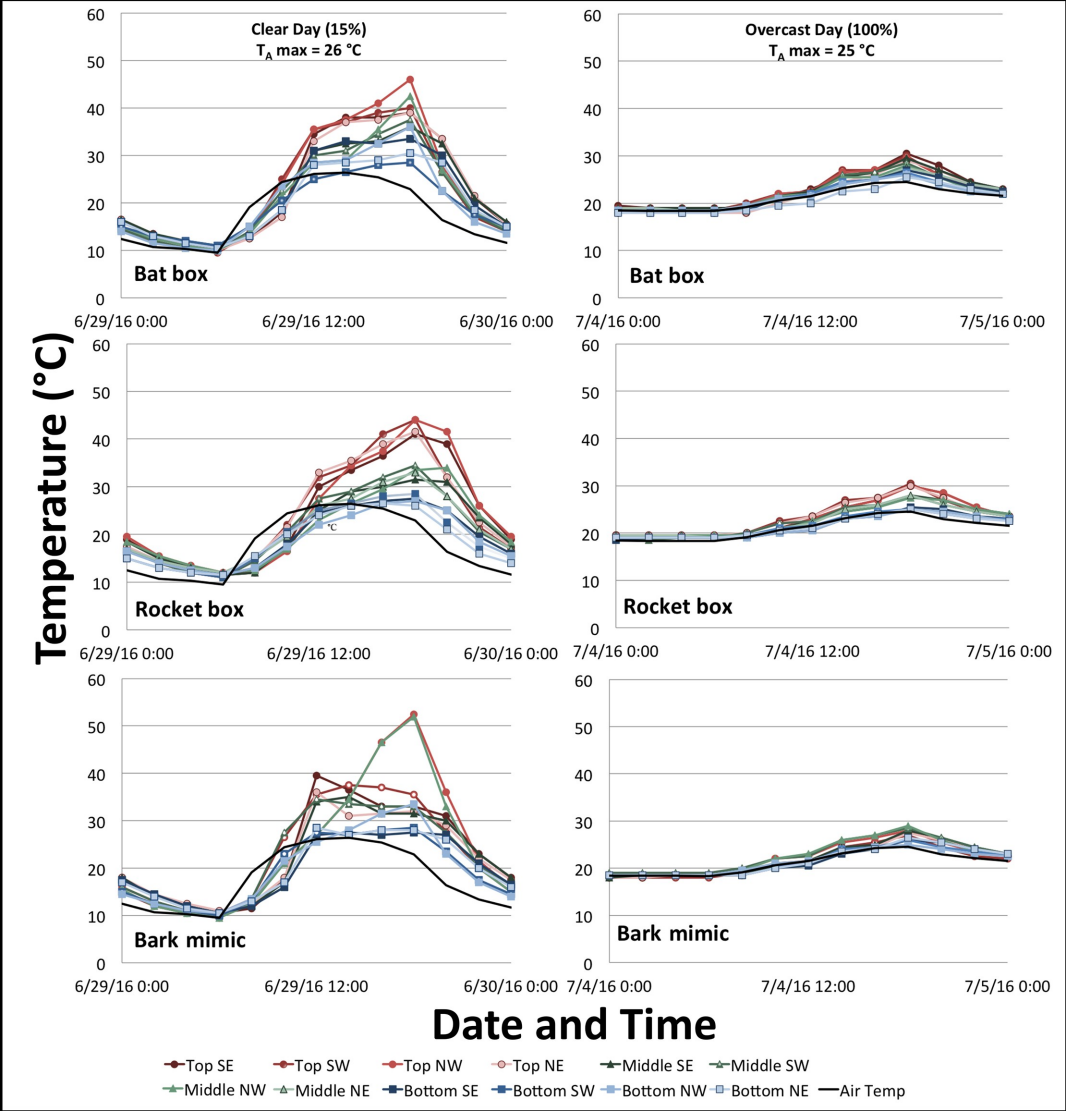
Two-day sample of temperatures recorded at each of 12 positions in artificial roosts. Weather station air temperature (black) recorded for two days in 2016 (29 June and 4 July), and same-day temperatures recorded by each of the iButton thermochrons in the bat box, rocket box, and bark mimic. Bats were excluded during temperature recording at this cluster of roosts in Plainfield, IN.

**Fig 3:**
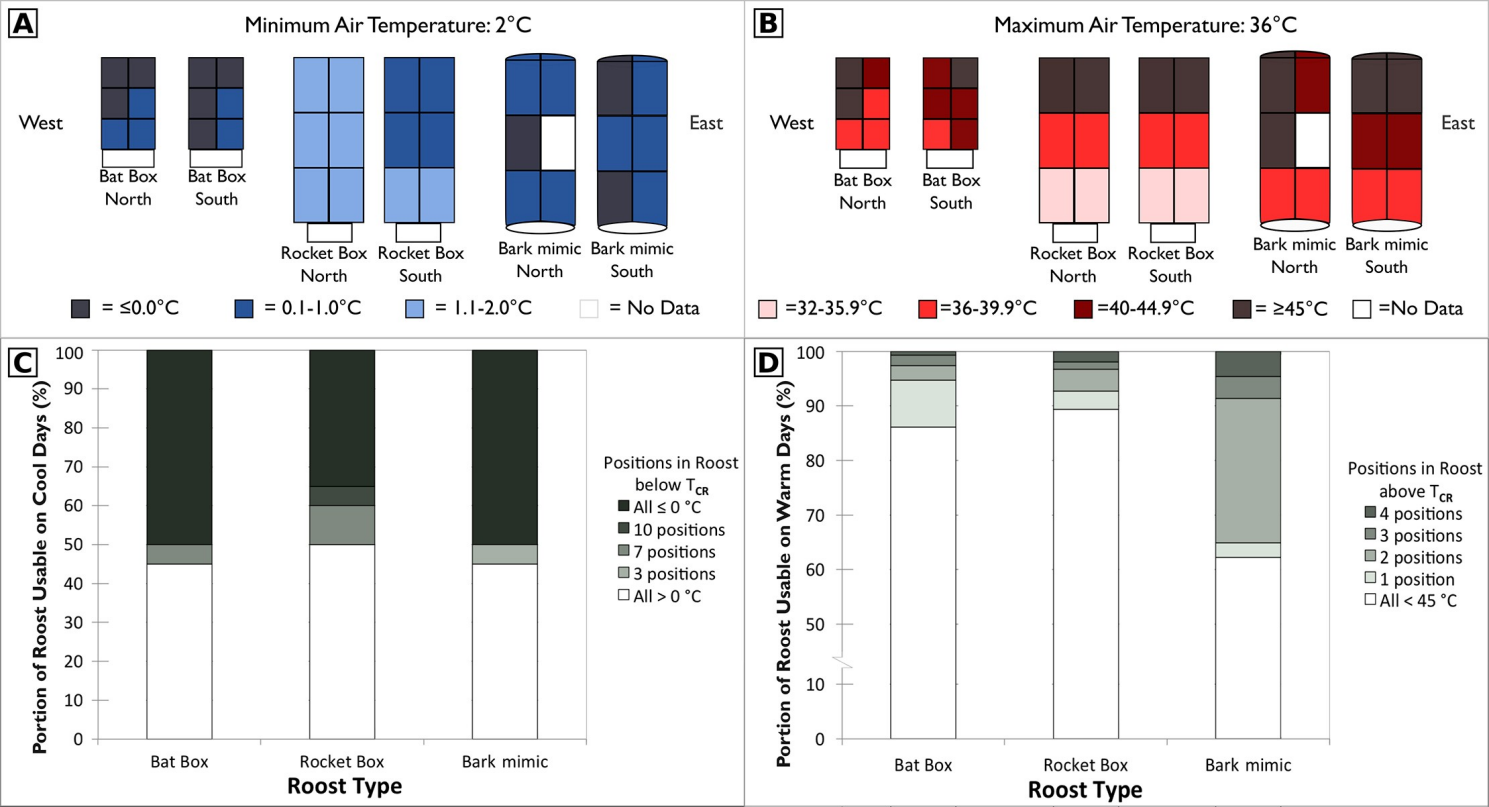
Temperatures recorded on sample cool and warm days, and portion of roost usable based on temperature data. Data loggers recorded temperature at three heights (top, middle, or bottom) and at four intercardinal directions (southeast, southwest, northwest, and northeast), as illustrated by the 12 sections for each roost (A and B). The data logger failed in the middle northeast of the bark-mimic roost. (A) Cool day example (16 May 2016) indicating minimum daily temperature recorded. (B) Warm day example (12 June 2016) indicating maximum daily temperature recorded. (C) Percent of cool days (mean air temperature < 10°C, n = 20 days) when portions of each roost type were ≤ 0°C and considered not usable. (D) Percent of warm days (mean air temperature ≥ 10°C, n = 151 days) when portions of each roost type were ≥ 45°C and considered not usable. Data collected at three adjacent artificial roosts from which bats were excluded, near Plainfield, IN, 21 March–7 September 2016.

In the bat-exclusion cluster, mean temperatures were 20–23°C across height levels, intercardinal directions, and roosts (S1 Table). Across roost types and positions, minimum temperatures ranged from −3 to −6°C and maximum temperatures ranged from 35–60°C (bat box max = 54°C, rocket box max = 51°C, and bark mimic max = 61°C; S1 Table and S1 Fig). Roost temperatures peaked after peak outside air temperature (Fig 2). On warm and clear days, the top portion of the rocket and bat box roosts could be > 10°C warmer than the bottom (Figs 2 and 3B), but tended to stay below T_CR_ (Fig 3D). However, on a warm day, points in both the top and middle positions in the bark mimic could rise above T_CR_ (Fig 3B), rendering larger proportions of the roost unusable (Fig 3D). On overcast days, temperatures inside the roosts more closely tracked outside air temperature, even in the middle of summer, and there was less variation among roost types or positions (Fig 2).

### Proportion usable

Across the 2016 season (21 March–7 September), a greater proportion of the roost remained within T_CR_ in the bat box (92%) and the rocket box (93%) than the bark mimic (87%). Temperatures below T_CR_ (0°C) were only recorded in the early part of the season—up to 13 April in the rocket box and 16 May in the bat box and bark mimic. With only 20 cool days (mean air temperature < 10°C; mean cool-day temperature 7 ± 1°C), we were unable to statistically assess cold-weather differences among roost types. Qualitatively, all roost types had similar responses to cool temperatures, with the rocket box buffering cool air temperatures only slightly better than the bat box or bark-mimic roost (Fig 3C). On a day when any one part of a roost measured below T_CR_, it was most often the case (84% of the time) that the entire roost would drop below T_CR_ (Fig 3C). Typically, we recorded temperatures ≤ 0°C for 3–11 hours at night or in early morning (mode = 7:00).

In contrast, on 151 warm days (mean air temperature ≥ 10°C; mean warm-day temperature 21 ± 0.5°C), regardless of roost type, no roost went entirely outside T_CR_ and the percent of the roost that was usable ranged from 64–100% (Fig 3D). However, on these warm days, a lower proportion of the bark-mimic roost was usable compared to the other roost types (Pseudo R^2^ = 0.24, p < 0.001); this effect was more pronounced with increasing Trange (p < 0.01). When a portion of the bark mimic was unusable, this was often due to excessively high temperatures inside the roost (mean maximum across 151 days was 40°C), typically recorded for 1–3 hours in the late afternoon or early evening (mode = 19:00).

### Availability

**Fig 4:**
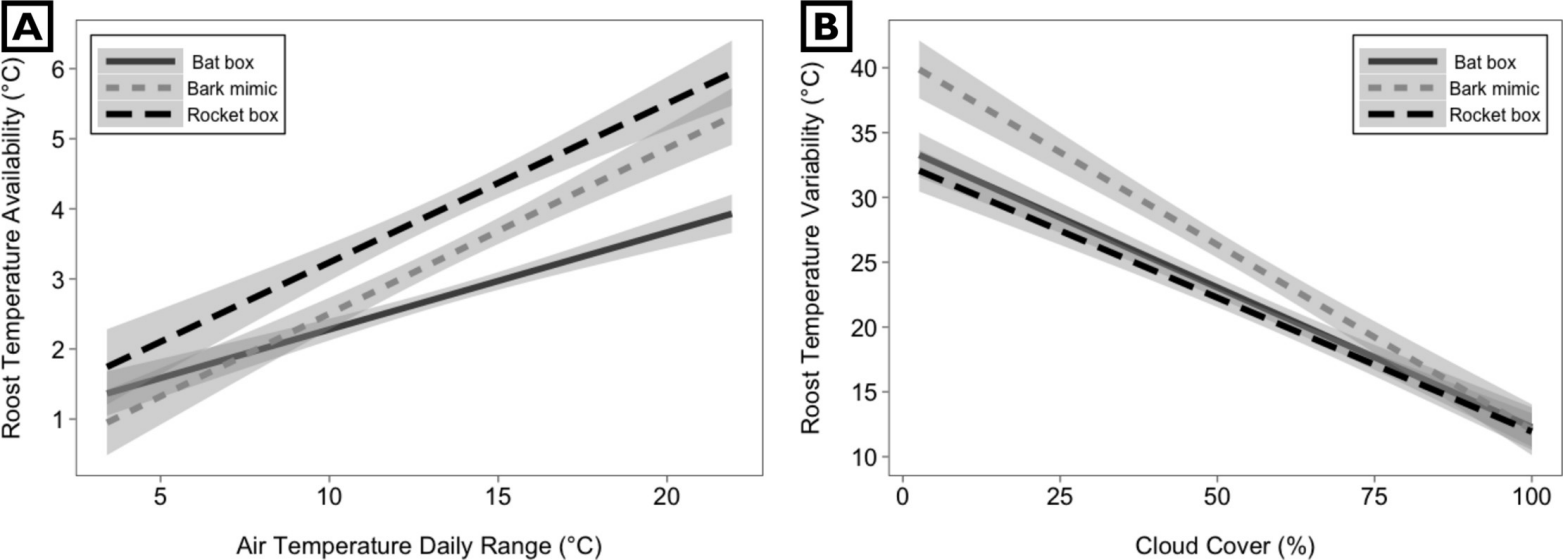
Interaction plots for predictor variables that significantly interacted with box type, with regression lines and 95% confidence intervals based on those interactions. (A) Roost temperature availability increased significantly with air temperature daily range (T_range_) and interacted with roost type (S2 Table). (B) Variability significantly decreased with increasing daily percent cloud cover, but this varied by roost type (S3 Table). Data collected from three adjacent artificial roosts from which bats were excluded, near Plainfield, IN.

After model simplification (see Methods), the accepted ANCOVA model to predict temperature availability included as main effects roost type, T_mean_, Trange, precipitation, percent cloud cover, and the 2-way interaction between roost type and Trange (multiple R^2^ = 0.60, residual SE = 0.94, F_8, 504_ = 93.46, p < 0.001; S2 Table). For all roost types, availability increased with increasing T_mean_ and Trange, and decreased with increasing precipitation and percent cloud cover (S2 Table). Across Trange, the rocket box roost provided wider availability than the bat box and bark mimic. However, the interaction between Trange and bat box temperature availability differed from interactions with the other two roost styles (p < 0.001, S2 Table). At Trange > 20°C, the modeled temperature availability in the bat box was nearly 1.5°C lower than the next closest roost type (Fig 4A).

### Variability

After model simplification (see Methods), the accepted ANCOVA model to predict roost temperature variability included as main effects roost type, cloud cover, Trange, precipitation, and the 2-way interaction between roost type and cloud cover (multiple R^2^ = 0.70, residual SE = 4.66, F_7, 505_ = 165.2, p < 0.001, S3 Table). For all roost types, variability increased with increasing Trange, and decreased with greater cloud cover and precipitation (S3 Table). On cloudy days, all roosts behaved similarly; however, on days with < 75% cloud cover, the bark-mimic roost was more variable than the rocket and bat box (Fig 4B). The bark mimic exhibited up to a 40°C range across a day with 0% cloud cover (p < 0.001; S3 Table and Fig 4B).

### Bat preference

In the five bat-selection clusters, the focal Indiana bat maternity colony repeatedly selected rocket boxes over bat boxes and bark-mimic roosts. Maximum weekly emergence count was significantly higher in the rocket boxes (p < 0.001, S4 Table). Maximum one night count was ~10 times larger in rocket boxes for both 2015 and 2016 (Table 3). The number of bat days in rocket boxes was 25–1,000 times higher than in bat boxes or bark-mimic roosts (Table 3). Of the five rocket boxes installed, four became primary maternity roosts, while we detected no bats during > 90% of counts at bat boxes and bark-mimic roosts (Table 3).

**Table 3:** Emergence and spot light counts (n = 2,214 counts) indicate the preference for rocket box style roosts in this maternity colony of Indiana bats.

Roost Type	2015 Max Count (n = 749)	2016 Max Count (n = 1,465)	2015 Total Bat Days^a^	2016 Total Bat Days^a^	Primary Roosts^b^ (> 30 bats)	No Bats (% Counts)
Bat box	22	7	172	24	0	90
Bark mimic	2	1	15	7	0	96
Rocket box	210	205	4,340	7,077	4	63

Data collected from three clusters in 2015 (9 roosts) and five clusters in 2016 (15 roosts) near Plainfield, IN, from March–September.

^a^One bat observed using the roost on one day

^b^[51]

## Discussion

Bats showed a clear preference by roost style, selecting the rocket box over bat box and bark-mimic roosts (Table 3). Notably, the rocket box style was the largest roost (i.e., volume, roosting surface area, and entrance area) and provided wide temperature availability while staying within T_CR_ the greatest proportion of time. The bat box was smaller and shorter; although availability was similar to the rocket box at low Trange, it had less usable space than the rocket box. The bark-mimic provided less usable space than the rocket box and, often, large proportions of the roost exceeded T_CR_. While all of these roost styles have documented use by Indiana bats [22,28,29] and are often used in mitigation, this is the first study to group these structurally-different roosts to assess microhabitat and selection. We cannot distinguish which of the characteristics of rocket boxes were the primary driver of roost selection, or if a combination of factors was at play because these common artificial roost designs differ with respect to multiple physical factors. We suggest additional empirical work exploring physical factors in isolation to determine which features are most critical to manipulate to achieve desirable conditions in artificial roosts.

Our focal colony selected the style with the largest volume, roosting surface area, and entrance area (Table 2), consistent with the tendency for Indiana bats to use larger diameter trees as primary roosts [18]. Many previous Indiana bat studies measured tree-roost diameter, height or percent exfoliating bark as proxies for roost size (see review [18]), but were unable to directly measure size characteristics. The rocket box was > 5 times larger in volume, > 2 times larger in roosting surface area, and > 2 times larger in roost entrance area than the other roost types (Table 2). Higher volume may have driven roost selection by providing space for group formation. For other tree-roosting bat species, cavity volume is positively correlated with roost selection, colony size, and roost use in consecutive years [53,54]. Roosting surface area may affect maternity colony size. For example, roost-tree diameter and colony sizes are lower for Indiana bats in the Appalachian Mountains [55] versus the Midwest [56]. A larger roost might also facilitate predator avoidance; bats often emerged from the rocket box in rapid succession at different roost aspects (pers. obs.), which could confuse predators or diffuse predation risk. More area from which to exit and 360° of possible exit points could facilitate predator avoidance [57], though, conversely, a narrow entrance gap may deter predators that climb the roost. We recommend additional work to compare artificial roosts that differ only with respect to volume, roost surface area, or entrance area to better understand the significance of available space to roost habitat selection.

The size of the roost is not the only determinant of usable space, as it is also important that within-roost temperatures are high enough to prevent freezing or excessive energy use [38] and stay below lethal limits [15,41]. In addition to selecting the largest roost style, Indiana bats selected the style in which the largest proportion stayed within the critical temperature limits (0–45°C; Fig 3). While bats prefer warm roosts [16,30], temperatures above their thermal neutral zone quickly become fatal [15]. Bats using roosts where temperatures exceed 45°C manage the heat behaviorally by moving to cooler areas within the roost [14,16]. The entire rocket box roost stayed within T_CR_ on 85% of all days measured (Fig 3). Greater portions of the smaller-volume bark-mimic roost reached very high temperatures (all middle and top positions reached ≥ 45°C) on warm days (≥ 1 position in the bark mimic exceeded T_CR_ 37% of the time on days where outside temperature was ≥ 10°C, Fig 3D). In such a roost, it may not be feasible for a large bat colony to find sufficient space at a manageable temperature across an entire warm day. However, usable proportion and peak temperatures might differ in a larger bark-mimic roost. We suggest additional work examining the role of construction material or ventilation in buffering high temperatures in upper sections of artificial roosts.

All roost styles were limited in their ability to buffer cool temperatures. Roost temperature is determined almost entirely by air temperature in cloudy conditions (Fig 2). Often the entire roost dropped below 0°C on cool days (< 10°C; Fig 3C) between 21 March and 16 May. To endure temperatures < 2°C, bats would have to expend energy to maintain a torpor threshold above freezing [15]. While Indiana bats may be more tolerant of such cold temperatures immediately following hibernation [15], roost designs that better buffer cold temperatures should be developed and evaluated, as such roosts may allow bats to save critical energy reserves; this may be especially important for bats recovering from white-nose syndrome [58].

The rocket box offered a wider range of available temperatures (S2 Table, Fig 4A). Bats using artificial roosts and buildings consistently select for wider temperature availability [16,25,30]. For example, little brown bats (*Myotis lucifugus)* select tall bat boxes, with wider temperature availability, over wide ones [30]. We found that as Trange increased, roost height promoted wider temperature availability. In both the tall rocket box and bark mimic (Table 2), a wide range of temperatures was possible at a given time (> 6°C). However, the rocket box consistently provided ~1°C wider temperature availability than the bark mimic (Fig 4A). Conversely, the shorter bat box showed a narrower range of temperatures at one time (< 5°C). Yet, both the rocket box and bat box were made of 3/4” (19 mm) wood boards, a good heat insulator that potentially trapped heat near the top of the roost at low T_mean_ and T_range_ (Fig 4A), and higher cloud cover (Fig 2), thereby providing a wider range of temperatures in certain conditions. Wider temperature availability likely allows bats to maintain preferred body temperature without expending energy for evaporative cooling, metabolic heat production, or moving to another roost [30,41]. We recommend future work manipulating artificial roost features that will affect temperature gradients; for example, it may be prudent to vary roost length, vent placement, size of roof overhang, and landscape position.

Solar radiation (cloud cover) exerted a significant effect on daily temperature variability (S3 Table), particularly on days with higher Trange. On clear days, we observed temperature peaks 10°C higher in the polyurethane bark mimic than in the wooden rocket and bat boxes (Fig 4B). Both material type and color can affect solar absorption, including absorption of color wavelength outside the visible range. All roost styles were similar in visible color (brown), but we did not test for color outside the visual range or pigment differences that may affect absorption. While hourly changes in roost temperatures may benefit bats by allowing them to passively rewarm after torpor [31], such variability could be detrimental when daily maxima exceed 45°C. Because variability was higher in the bark-mimic roost on clear days (Figs 2 and 4B), we recommend future studies evaluate temperature variability in bark-mimic roosts when positioned under greater canopy cover or painted a lighter color.

While we found a clear selection by roost type (Table 3), undoubtedly the result of a combination of underlying factors, we did not isolate structural and microhabitat differences in this study, and assessed only one colony of one species. Factors such as group formation [59,60], predation risk [57], parasite loads in the roost [30], roost familiarity, and regional preferences (e.g., [55]) may also contribute to roost preferences. We advise replicating this study with structures that isolate important factors affecting size and microclimate, and recommend exploring roost microhabitat preferences for multiple colonies and bat species.

Roosts are crucial to bat survival and, with increasing development pressures, knowledge about roost preference is essential to creating better artificial roosts and protecting preferred natural roosts. Artificial roosts may provide an immediate alternative for displaced bat colonies [30], and may mitigate for the loss of natural roosts in some areas (e.g. [22,34]). While providing artificial roosts may be merited in areas where few suitable roost trees are present, artificial roosts may not be adequate surrogates for natural roosts, and are not a panacea for the overall loss of roosting and foraging habitat [34]. Researchers and managers must be careful not to use artificial roosts in a cavalier fashion. Microclimates in artificial roosts differ in their cyclical fluctuations when compared to natural roosts [22], and may hinder bats’ fitness and survival if artificial roost microclimates are too hot [30] or too cold [58]. It is fairly uncommon for researchers to use datalogging devices to measure microclimates in natural roosts, though such devices provide a detailed picture of the spatial and temporal variation in roost microclimate [61]. Our comparative assessment of the microclimates of artificial roosts allowed us to account for some of the inherent irregularity in natural systems and to identify factors important to roost microhabitat selection. Parameters likely to be important for roost selection by Indiana bats include large size, temperatures that stay within T_CR_ (0–45°C), and consistently wide availability of temperatures. We suggest managers proceed with caution when substituting artificial roosts for natural habitat, taking season-long microclimate measurements inside novel artificial roosts [61] before making them available to bats. Furthermore, we recommend research to compare use of natural versus artificial roosts, assess impacts of landscape and climate on artificial roost use, and develop and compare new artificial roost styles for bats that are imperiled due to loss of optimal roosting habitat.

## Supporting information

S1 Table Mean temperatures by position. Mean ± SD (range) of temperatures (°C) recorded from 21 March–7 September 2016 by each iButton thermochron positioned throughout three adjacent artificial roosts (bat box, rocket box, and bark mimic) where bats were excluded.
(DOCX)

S2 Table Model results for availability. Model results (parameter estimate, standard error, t value, and p value) of the most parsimonious analysis of covariance describing the effects of weather parameters on mean hourly temperature availability with the covariate of roost type (bark mimic, bat box, and rocket box).
(DOCX)

S3 Table Model results for variability. Model results (parameter estimate, standard error, t value, and p value) from an analysis of covariance of weather parameters on daily roost temperature variability with the covariate of roost type (bark mimic, bat box, and rocket box).
(DOCX)

S4 Table Model results for bat preference. Model results (random and fixed effects) from the generalized linear mixed model (GLMM) of the maximum weekly emergence count by roost type (bark mimic, bat box, and rocket box) with cluster as a random effect.
(DOCX)

S1 Fig Boxplots of temperature by position. Temperatures (°C) recorded from 21 March–7 September 2016 by each iButton thermochron positioned throughout three adjacent artificial roosts (bat box, rocket box, and bark mimic) where bats were excluded. Position indicated by three-letter code: height (B = bottom, M = middle, T = top) and intercardinal direction (NE = northeast, NW = northwest, SE = southeast, SW = southwest).
(DOCX)
