# Supplementary material for: In artificial roost comparison, bats show preference for rocket box style
Source: PLoS One. 2018 Oct 31;13(10):e0205701. doi: 10.1371/journal.pone.0205701 (PMC6209394; doi:10.1371/journal.pone.0205701)
Supplement: S1 Table — Mean ± SD (range) of temperatures (°C) recorded from 21 March–7 September 2016 by each iButton thermochron positioned throughout three adjacent artificial roosts (bat box, rocket box, and bark mimic) where bats were excluded. (DOCX) [file pone.0205701.s001.docx]

# PLOS One Supporting Information

In artificial roost comparison, bats show preference for rocket box style

Julia P. S. Hoeh, George S. Bakken, William A. Mitchell, Joy M. O’Keefe^*^

**S1 Table. Mean temperatures by position.** Mean ± SD (range) of temperatures (°C) recorded from 21 March–7 September 2016 by each iButton thermochron positioned throughout three adjacent artificial roosts (bat box, rocket box, and bark mimic) where bats were excluded. At each height within the roost (top, middle, or bottom), four thermochron data loggers were attached in mesh to the roost wall at four intercardinal directions (southeast, southwest, northwest, and northeast). Data collected near Plainfield, IN.

| Height | Direction | Bat box | Rocket box | Bark mimic |
| --- | --- | --- | --- | --- |
| Top | SE | 22 ± 10 (−5–45) | 23 ± 10 (−5–48) | 22 ± 10 (−5–48) |
|  | SW | 22 ± 10 (−6–48) | 23 ± 10 (−5–50) | 22 ± 10 (−6–51) |
|  | NW | 22 ± 11 (−6–54) | 23 ± 10 (−5–51) | 23 ± 11 (−5–61) |
|  | NE | 22 ± 10 (−5–45) | 23 ± 10 (−4–48) | 21 ± 9 (−5–45) |
| Middle | SE | 22 ± 9 (−4–43) | 21 ± 9 (−5–41) | 22 ± 10 (−5–45) |
|  | SW | 21 ± 9 (−5–45) | 21 ± 9 (−5–42) | 22 ± 10 (−5–45) |
|  | NW | 21 ± 9 (−6–49) | 21 ± 9 (−4–40) | 22 ± 11 (−5–60) |
|  | NE | 21 ± 9 (−4–40) | 21 ± 9 (−4–39) | –a |
| Bottom | SE | 21 ± 9 (−4–41) | 20 ± 8 (−3–36) | 20 ± 8 (−4–37) |
|  | SW | 21 ± 9 (−5–43) | 20 ± 8 (−4–37) | 20 ± 8 (−5–38) |
|  | NW | 20 ± 9 (−5–43) | 20 ± 8 (−3–35) | 20 ± 8 (−4–42) |
|  | NE | 20 ± 8 (−4–38) | 20 ± 8 (−3–35) | 20 ± 8 (−4–37) |

aData logger failed to record.
