# Supplementary material for: In artificial roost comparison, bats show preference for rocket box style
Source: PLoS One. 2018 Oct 31;13(10):e0205701. doi: 10.1371/journal.pone.0205701 (PMC6209394; doi:10.1371/journal.pone.0205701)
Supplement: S2 Table — Model results (parameter estimate, standard error, t value, and p value) of the most parsimonious analysis of covariance describing the effects of weather parameters on mean hourly temperature availability with the covariate of roost type (bark mimic, bat box, and rocket box). (DOCX) [file pone.0205701.s002.docx]

# PLOS One Supporting Information

In artificial roost comparison, bats show preference for rocket box style

Julia P. S. Hoeh, George S. Bakken, William A. Mitchell, Joy M. O’Keefe^*^

S2 Table. Model results for availability. Model results (parameter estimate, standard error, t value, and p value) of the most parsimonious analysis of covariance describing the effects of weather parameters on mean hourly temperature availability with the covariate of roost type (bark mimic, bat box, and rocket box). Data were collected from three adjacent artificial roosts near Plainfield, IN where bats were excluded March–September 2016.

| Parameter | Estimate | SE | t | p |
| --- | --- | --- | --- | --- |
| Bark mimic | 2.71 | 0.36 | 7.57 | < 0.001 |
| Bat box | 0.75 | 0.36 | 2.11 | < 0.05 |
| Rocket box | 0.83 | 0.36 | 2.33 | < 0.05 |
| T**mean** | 0.05 | 0.01 | 6.63 | < 0.001 |
| T**range** | 0.09 | 0.02 | 4.31 | < 0.001 |
| Precipitation | −0.02 | 0.01 | −4.42 | < 0.001 |
| % Cloud Cover | −2.56 | 0.23 | −11.29 | < 0.001 |
| Bat box: T**range** | −0.10 | 0.03 | −3.86 | < 0.001 |
| Rocket box: T**range** | −0.01 | 0.03 | −0.37 | 0.71 |
