# Supplementary material for: In artificial roost comparison, bats show preference for rocket box style
Source: PLoS One. 2018 Oct 31;13(10):e0205701. doi: 10.1371/journal.pone.0205701 (PMC6209394; doi:10.1371/journal.pone.0205701)
Supplement: S3 Table — Model results (parameter estimate, standard error, t value, and p value) from an analysis of covariance of weather parameters on daily roost temperature variability with the covariate of roost type (bark mimic, bat box, and rocket box). (DOCX) [file pone.0205701.s003.docx]

# PLOS One Supporting Information

In artificial roost comparison, bats show preference for rocket box style

Julia P. S. Hoeh, George S. Bakken, William A. Mitchell, Joy M. O’Keefe^*^

S3 Table. Model results for variability. Model results (parameter estimate, standard error, t value, and p value) from an analysis of covariance of weather parameters on daily roost temperature variability with the covariate of roost type (bark mimic, bat box, and rocket box). Data collected from three adjacent artificial roosts near Plainfield, IN where bats were excluded March–September 2016.

| Parameter | Estimate | | SE | | t | p |
| --- | --- | --- | --- | --- | --- | --- |
| Bark mimic | 23.32 | 1.58 | | 14.74 | | < 0.001 |
| Bat box | −6.78 | 1.19 | | −5.71 | | < 0.001 |
| Rocket box | −8.01 | 1.19 | | −6.75 | | < 0.001 |
| % Cloud Cover | −17.66 | 1.58 | | −11.18 | | < 0.001 |
| T**range** | 0.88 | 0.07 | | 12.62 | | < 0.001 |
| Precipitation | −0.12 | 0.02 | | −5.27 | | < 0.001 |
| Bat box: % Cloud Cover | 6.98 | 1.94 | | 3.59 | | < 0.001 |
| Rocket box: % Cloud Cover | 7.86 | 1.94 | | 4.05 | | < 0.001 |
