# Supplementary material for: In artificial roost comparison, bats show preference for rocket box style
Source: PLoS One. 2018 Oct 31;13(10):e0205701. doi: 10.1371/journal.pone.0205701 (PMC6209394; doi:10.1371/journal.pone.0205701)
Supplement: S4 Table — Model results (random and fixed effects) from the generalized linear mixed model (GLMM) of the maximum weekly emergence count by roost type (bark mimic, bat box, and rocket box) with cluster as a random effect. (DOCX) [file pone.0205701.s004.docx]

# PLOS One Supporting Information

In artificial roost comparison, bats show preference for rocket box style

Julia P. S. Hoeh, George S. Bakken, William A. Mitchell, Joy M. O’Keefe^*^

S4 Table. Model results for bat preference. Model results (random and fixed effects) from the generalized linear mixed model (GLMM) of the maximum weekly emergence count by roost type (bark mimic, bat box, and rocket box) with cluster as a random effect. Data collected from five clusters of three adjacent artificial roosts (15 total roosts) near Plainfield, IN from March–October 2015–2016.

| Random effects: | |  |  |  |
| --- | --- | --- | --- | --- |
| Groups | Name | Variance | SD |  |
| Cluster | (Intercept) | 1.197 | 1.094 |  |
| Number of observations: 725, groups: Cluster, 5 | | | | |
|  |  |  |  |  |
| Fixed effects: |  |  |  |  |
| Variable | Estimate | SE | z | p |
| Bark mimic | −2.88 | 0.60 | −4.78 | < 0.001 |
| Bat box | 1.35 | 0.43 | 3.16 | < 0.01 |
| Rocket box | 5.14 | 0.38 | 13.49 | < 0.001 |
