# Supplementary material for: In artificial roost comparison, bats show preference for rocket box style
Source: PLoS One. 2018 Oct 31;13(10):e0205701. doi: 10.1371/journal.pone.0205701 (PMC6209394; doi:10.1371/journal.pone.0205701)

# PLOS One Supporting Information

In artificial roost comparison, bats show preference for rocket box style

Julia P. S. Hoeh, George S. Bakken, William A. Mitchell, Joy M. O’Keefe^*^

S1 Figure. Boxplots of temperature by position. Temperatures (°C) recorded from 21 March–7 September 2016 by each iButton thermochron positioned throughout three adjacent artificial roosts (bat box, rocket box, and bark mimic) where bats were excluded. Position indicated by three-letter code: height (B=bottom, M=middle, T=top) and intercardinal direction (NE=northeast, NW=northwest, SE=southeast, SW=southwest).


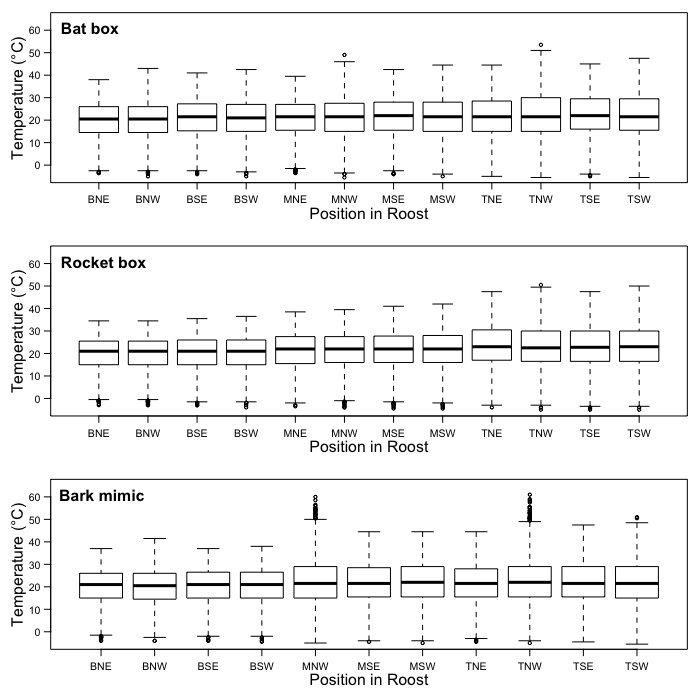

Supplement: S1 Fig — Temperatures (°C) recorded from 21 March–7 September 2016 by each iButton thermochron positioned throughout three adjacent artificial roosts (bat box, rocket box, and bark mimic) where bats were excluded. Position indicated by three-letter code: height (B = bottom, M = middle, T = top) and intercardinal direction (NE = northeast, NW = northwest, SE = southeast, SW = southwest). (DOCX) [file pone.0205701.s005.docx]
